# Development and Validation of a Pioneer Scale on Service Leadership Behavior in the Service Economies

**DOI:** 10.3389/fpsyg.2019.01770

**Published:** 2019-08-02

**Authors:** Daniel T. L. Shek, Diya Dou, Lawrence K. Ma

**Affiliations:** ^1^Department of Applied Social Sciences, The Hong Kong Polytechnic University, Kowloon, Hong Kong; ^2^Department of Psychology, The Education University of Hong Kong, Tai Po, Hong Kong

**Keywords:** scale validation, service leadership, leadership education, confirmatory factor analysis, Hong Kong

## Abstract

In response to the severe lack of leadership assessment tools in the Chinese context, the Service Leadership Behavior Scale was developed based on the Service Leadership Model proposed by Po Chung, the co-founder of DHL International. Utilizing responses from 4,486 Hong Kong undergraduates, this paper reports the findings of a validation study on the Short-Form Service Leadership Behavior Scale (SLB-SF-65). Previous findings based on exploratory factor analysis supported a six-factor 48-item solution (SLB-SF-48). With the removal of ten items, confirmatory factor analysis showed that the final 38-item scale (SLB-SF-38) possessed excellent internal consistency, concurrent validity, and factorial validity based on multigroup invariance analyses. Overall speaking, the present study underscores the utility of the SLB-SF-38 as an objective assessment instrument of service leadership behavior in the education, research and personnel training contexts.

## Introduction

Over the past few decades, a structural transformation from the manufacturing-based to service-focused economies has been observed in many developed as well as developing countries (Bryson and Daniels, 2015; Snell et al., 2017). As such, possessing effective leadership qualities in this service era is indispensable in the contemporary world (Chung, 2015; Chung and Elfassy, 2016).

This service-focused leadership has been widely discussed in literature on both public and commercial service units. According to Schneider et al. (2005), leader’s service-focused behavior, or service leadership, communicates a commitment to high levels of service quality. Compared with general leadership, service leadership is believed to exert a stronger influence on service outcomes (Hong et al., 2013). It is argued that service-oriented management and effective service leadership foster a service climate and consequently improve service performance (Jiang et al., 2015). Some assessment tools on service leadership have been developed and adopted in related empirical studies (Schneider et al., 2005; Jiang et al., 2015), such as Service Climate Scale (includes items measuring service-oriented leadership behavior) developed by Schneider et al. (1998), and a managerial measure of organizational service-orientation developed by Lytle et al. (1998), where service leadership was conceptualized as a combination of servant leadership and service orientation.

Although available scales measuring service leadership have a solid theoretical foundation and engendered much research, some research gaps exist. First, these scales were often developed with a strong focus on customer service. However, “service” in service economy should be interpreted in a broader context involving not only customer service but also the commitment to self-development, service to followers as well as society. Second, although service leadership is closely related to servant leadership, they are distinct concepts (Sendjaya and Sarros, 2002; Wong et al., 2015). According to the servant leadership theory, followers’ needs precede leaders’ individual needs (Shek et al., 2015a). In contrast, service leadership seeks the mutual satisfaction of needs of both leaders and followers. Therefore, servant leadership scales may not be totally appropriate to assess service leadership. Third, available scales of service leadership mainly focus on leadership competences that guide and reward service delivery (i.e., “doing” of service leadership), such as goal setting, planning and coordinating (Schneider et al., 2005). Leaders’ ability to make moral decisions and caring for others (i.e., “being” of service leadership) have often been considered relevant factors but not indispensable attributes of service leadership (Jiang et al., 2016). To fill the gaps, a set of assessment tools measuring service leadership was developed based on the Service Leadership Model proposed by Po Chung (Shek et al., 2015b, 2018a). In the following parts, the Service Leadership Model, its unique features, and the project entailing the construction and validation of Service Leadership Scales are outlined.

### The Service Leadership Model and Its Unique Features

Service leadership is conceptualized as a “service aimed at ethically satisfying the need of self, others, groups, communities, systems, and environments” (Shek and Lin, 2015a, p. 233). The Service Leadership Model highlights three core attributes: *Competence*, *Character*, and *Caring*. First, *Competence* covers one’s task-specific knowledge and skill sets required to excel in operational duties, which are essential for leaders to win over their followers (Chung and Bell, 2015). *Character* is defined as one’s propensity to behave “in ways that are consistent with high [moral] values” (Chung and Elfassy, 2016, p. 59), to command respect and trust from followers. *Care* entails harboring an unselfish intent toward others so as facilitating their growth and development (Greenleaf, 1977; Shek and Li, 2015).

The Service Leadership Model builds on and complements other existing leadership paradigms such as servant leadership, ethical leadership, and transformational leadership (see Shek et al., 2015a for a thorough review). First, as discussed earlier, contrary to the servant leadership model deemphasizing one’s own needs (Greenleaf, 1970; Russell and Stone, 2002), effective service leadership appreciates self-serving endeavors to develop one’s capacity and eagerness to satisfy others’ needs. Second, while the ethical leadership model emphasizes moral *Character* (Brown and Treviño, 2006), *Competence* (Shek et al., 2015a) and service provision on the “self” and “others” levels (Mendonca, 2001), how *Care* impacts leadership effectiveness remains under-addressed (Shek et al., 2015a). Third, transformational leaders motivate the pursuit of collective goals at the expense of personal interest, and in so doing these leaders help followers fulfill their potential through idealized influence, inspirational motivation, intellectual stimulation, and individualized considerations (Bass, 1990; Avolio et al., 1999). Transformational leadership theory has limited coverage on *Competence* and *Care* as the determinants of leadership success (Shek et al., 2015a).

In a nutshell, the Service Leadership Model incorporates several core features of related leadership paradigms and attempts to build up an integrative perspective in leadership (Shek et al., 2015a). Such a perspective inspires the education of a generation of new leaders that can thrive in this service era (Shek and Chung, 2015; Shek et al., 2015c, 2017).

### Service Leadership Education in Hong Kong

As one of the most important outcomes of higher education, leadership of university students is highly regarded by both universities and employers (Bacon et al., 1979). However, a discrepancy exists between employers’ expectation and what university students could demonstrate in service economies (Shek et al., 2017). Such a discrepancy results in a mismatch in recruitment, low job satisfaction and even mental burnout amongst the existing staff (Towers Watson, 2012). Thus, Po Chung, the co-founder of DHL International and the incumbent chairperson of the Hong Kong Institute of Service Leadership & Management Limited (HKI-SLAM), put forth the Service Leadership Model with a vision to nurture a generation of emergent service leaders who are not only competent, but are also moral and caring (Shek et al., 2017).

To promote quality leadership education conducive to students’ personal growth and employability, Chung argued passionately for the need to incorporate formal training based on the Service Leadership Model into the curriculum of undergraduates in Hong Kong (Chung, 2015; Shek et al., 2015c). With the financial support of the Victor and William Fung Foundation and the collaborative effort from the HKI-SLAM and universities financed by the University Grants Committee (UGC), a multi-year project entitled “Fung Service Leadership Education Initiative (FSLEI)” was implemented in eight UGC-funded universities in Hong Kong. Based on the Service Leadership and Management (SLAM) curriculum framework proposed by the Hong Kong Institute of Service Leadership and Management Limited [HKI-SLAM] (2013), all institutions under the FSLEI independently developed programs and curriculum materials that facilitate learning of service leadership at the undergraduate level (Shek and Chung, 2015). While it is important to develop service leadership curriculum materials and training programs, it is equally important to develop objective measures of service leadership qualities (Shek and Chung, 2015). Unfortunately, the paucity of validated assessment tools on service leadership in the Chinese context (Shek et al., 2017) has hindered meaningful analyses on the effectiveness of service leadership education under the FSLEI (Shek and Lin, 2015b, 2017).

Against such a backdrop, the research team at a Hong Kong university initiated a multi-year project entitled ‘Development and validation of measures based on the Service Leadership Model’ (Shek et al., 2017). This project entailed the construction and validation of three scales, each of which constituted a parameter of success of an educational program (Shek and Lin, 2017) pertaining to one’s *Attitude*, *Behavior*, and *Knowledge* on the Service Leadership Model (Shek et al., 2017). Some related publications can be seen elsewhere (e.g., Shek et al., 2018b,c,f; Shek and Chai, 2019). This paper primarily discusses the findings of a large-scale validation study on the Service Leadership Behavior Scale, which was designed to measure one’s exhibited behavioral attributes characteristic of a service leader.

### Service Leadership Behavior Scale

As part of the research program (Shek et al., 2017), the Long-Form Service Leadership Behavior Scale (SLB-LF-97) was developed primarily based on the SLAM curriculum framework (Hong Kong Institute of Service Leadership and Management Limited [HKI-SLAM], 2013), *25 Principles of Service Leadership* (Chung and Bell, 2015), *12 dimensions of a Service Leader* (Chung and Elfassy, 2016), and other published works from the leadership literature (e.g., Wielkiewicz, 2000; Ho and Nesbit, 2009). Initially, the SLB-LF-97 contained the following proposed domains: 3-Cs model (*Competence*, *Character* and *Care*), service provision, commitment to continuous improvement, and distributed leadership.

The SLB-LF-97 was administered in a preliminary validation study involving 231 university students (Shek et al., 2018b), where the results informed the retention of 65 items forming a short-form of the scale (SLB-SF-65). The SLB-SF-65 included 12 factors: problem-solving, self-leadership and life-long learning, non-cognitive intrapersonal competences, distributed leadership, integrity, care provision, concern, self-reflection, service provision, positive social relationship, communication skills, and fairness (Shek et al., 2018b). Both the SLB-LF-97 and the SLB-SF-65 exhibited excellent reliability (αs > 0.95) and robust convergent validity, with the latter evidenced by the significant and positive correlation with a host of theoretically relevant constructs such as servant leadership (*r* = 0.78) and leadership self-efficacy (*r* = 0.55) (Shek et al., 2018c). Nonetheless, the dimensionality of the SLB-SF-65 remained to be ascertained owing to the relatively modest sample size (*N* = 231). The background, conceptual model and steps involved in the development of different forms of Service Leadership Behavior Scales are outlined in Shek et al. (2018e).

### Objectives of the Present Study

Utilizing the data from a validation study involving 4,486 undergraduates from eight UGC-funded universities, the present study sought to build upon the abovementioned preliminary validation study (Shek et al., 2018c) in two ways. First, following the commonly adopted two-step dimensionality analysis (Park, 2014; Besnoy et al., 2016) involving an exploratory factor analysis (EFA) followed by a confirmatory factor analysis (CFA), the present study attempted to examine the dimensionality of the SLB-SF-65. Second, via the utilization of a much larger sample alongside several well-validated external criterion measures adopted in the study of Shek et al. (2018c), the present study attempted to further establish the reliability and convergent validity of the SLB-SF-65. Based on Shek et al.’s (2018c) initial findings, this study constituted a pioneer effort to construct and validate an objective assessment tool on service leadership in a Chinese context. The present findings contribute to the scanty literature of service leadership evaluation in the Chinese context (Shek and Lin, 2015b, 2017) and serve to produce a valuable instrument to assess learning outcomes of service leadership training programs (Shek and Chung, 2015).

**FIGURE 1:**
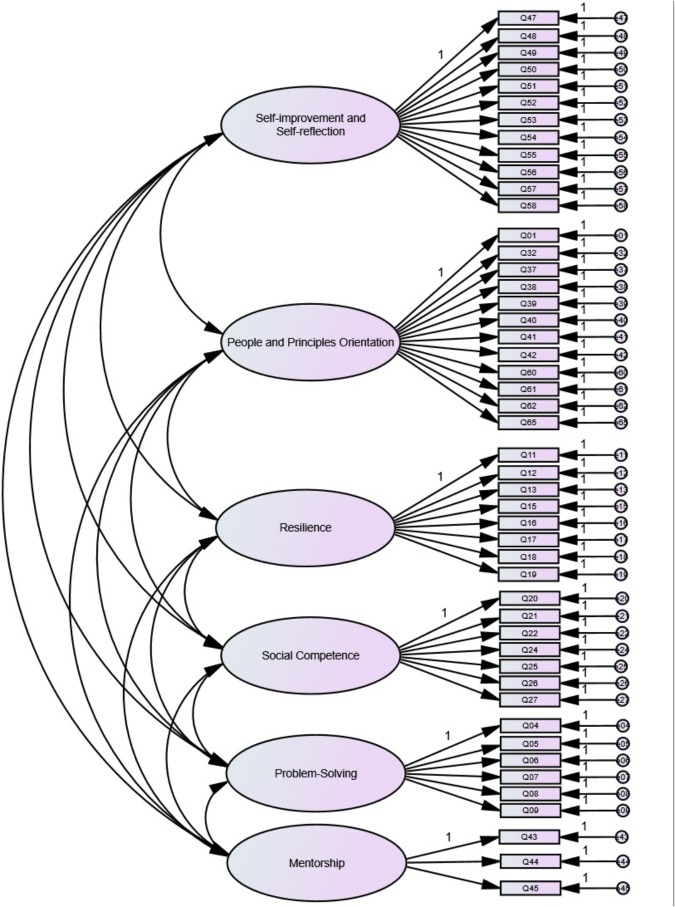
The initial six-factor, 48-item factorial structure (Model 0; i.e., SLB-SF-48).

In the present study, evaluation of factorial validity of the SLB-SF-65 involved two steps, with the dataset (*N* = 4,486) randomly split into two halves (subsets A and B) to facilitate both the EFA and the CFA. The EFA performed on subset A (*N* = 2,246) resulted in a stable and valid initial six-factor, 48-item solution (SLB-SF-48, see Figure 1), which was consistent with the original conceptual model. Details pertaining to the EFA were reported in Shek et al. (2018c). The six factors, each of which formed a subscale on the basic dimensions of service leadership, were accordingly named (a) Self-improvement and Self-reflection (12 items), (b) People and Principles Orientation (12 items), (c) Resilience (8 items), (d) Social Competence (7 items), (e) Problem-Solving (6 items), and (f) Mentorship (3 items). In this paper, this six-factor solution was then subjected to a CFA performed on subset B (*N* = 2,240), with the objective to evaluate how this proposed model fit the rest of the data and stability of the factor structure.

## Materials and Methods

The data were derived from a research project on service leadership involving eight UGC-funded universities in Hong Kong. Students were invited to participate in the survey via an electronic platform. The data were collected between March and June, 2017. During the survey, the purpose of this study, the principles of voluntary participation and withdrawal, and the compensation arrangement were explained on the survey webpage and the invitation documents. Students were asked to indicate their acceptance or refusal to join the study on the opening page. We rewarded each participant a supermarket gift voucher valued at HK$100 (US$12.80).

### Procedures

In total, 4,555 completed responses were retrieved. Three steps were performed for data cleaning. First, we removed six cases in which students declined to participate. Second, 30 cases were excluded because either they had completed the questionnaire designed for universities other than their own, or they revealed themselves as non-undergraduates in open-ended questions. Third, after reviewing respondents’ student identity number (which is anonymous to the Research Team), 33 cases with multiple participation were removed from the sample. Ultimately, 4,486 cases were retained as the working sample.

### Profiles of the Respondents

Among the 4,486 students, 1,517 were males and 2,969 were females. The majority of the sample were aged 20–24 years (68.4%; mean age = 20.47 years, *SD* = 1.67), had previous work experience (91.4%), and assumed the leadership position before (61.4%). Most participants had not received credit- or non-credit-bearing training in service leadership before (74.3 and 82.0%, respectively), and claimed to know “a little” or “some” about service leadership (75.0%).

### Instruments

#### Assessment of Service Leadership Qualities

The Long-Form Service Leadership Behavior Scale (SLB-LF-97) was designed to measure the behavioral attributes of an effective service leader (Shek et al., 2017). The 97 scale items were developed based on the general leadership literature (e.g., Wielkiewicz, 2000; Ho and Nesbit, 2009), publications based on the Service Leadership Model (e.g., Chung and Bell, 2015; Shek et al., 2015c; Chung and Elfassy, 2016) and the SLAM curriculum framework (Hong Kong Institute of Service Leadership and Management Limited [HKI-SLAM], 2013), with four domains, including the 3-Cs model (*Competence*, *Character* and *Care*), service provision, commitment to continuous improvement, and distributed leadership. The SLB-LF-97 was validated in a study involving 231 students from a university in Hong Kong (Shek et al., 2018b). The findings suggested the retention of 65 items to form the SLB-SF-65, which was employed in the present study. The dimensions derived are generally consistent with the original conceptual model. Each item of the SLB-SF-65 describes a specific leadership behavior where the respondents evaluate how well each item describes their leadership behavior (see Table 1 for sample items). A six-point Likert scale was used (1 = very dissimilar; 6 = very similar). Both the SLB-LF-97 and the SLB-SF-65 recorded excellent internal consistency (αs > 0.95; mean inter-item correlations > 0.25) in the previous validation study (Shek et al., 2018c).

**Table 1:** Sample items of the Short-Form Service Leadership Behavior Scale (SLB-SF-65).

Items	Very Dissimilar to Me	Moderately Dissimilar to Me	Slightly Dissimilar to Me	Slightly Similar to Me	Moderately Similar to Me	Very Similar to Me
Sample item 1. I try to serve others without regard to their positions.	1	2	3	4	5	6
Sample item 2. I refuse to give in without a fight amidst adversity.	1	2	3	4	5	6
Sample item 3. I have no problem working with others.	1	2	3	4	5	6

*All sample items were slightly re-phrased to avoid practice effect.*

The research also entailed the construction of scales designed to assess individuals’ knowledge of the Service Leadership Model (Shek et al., 2017, p. 167) as well as their attitudes and beliefs about desired leadership qualities (Shek et al., 2017, p. 212). In the present study, the shortened final versions of these two scales were administered.

#### Short-Form Service Leadership Knowledge Scale (SLK-SF-40)

The Service Leadership Knowledge Scale was developed based on the SLAM curriculum framework (Hong Kong Institute of Service Leadership and Management Limited [HKI-SLAM], 2013) and the literature on service leadership (e.g., Shek et al., 2015c; Chung and Elfassy, 2016). Participants’ responses to the original 200 items were coded based on accuracies (1 = correct; 0 = incorrect). Based on a criterion-validation study involving 160 Hong Kong university students (Shek and Lin, 2017), 50 items were retained to form the shortened scale (SLK-SF-50). Then the SLK-SF-50 was administered in a large-scale validation study, of which the results suggested the removal of additional 10 items to form the final SLK-SF-40 (Shek et al., 2018d). Table 2 illustrates several sample items of the final SLK-SF-40 administered in the present validation study.

**Table 2:** Sample items of the Short-Form Service Leadership Knowledge Scale (SLK-SF-40).

Items	Options	Correct answer
Sample item 1: A manager under the service economy wants to hire someone. Based on the Service Leadership Model, which of the following advice would you give him/her?	(A) Hire for qualifications, train for character (B) Hire for character, train for skills (C) Hire for attitude, train for character (D) Hire for efficiency, train for mindset	B
Sample item 2: Meg devoted herself to a career in relieving people of their hunger, isolation, and poverty. Which dimension of character strengths was shown by Meg’s devotion?	(A) Justice (B) Courage (C) Humanity (D) Temperance	C

*All sample items were slightly re-phrased to avoid practice effect.*

#### Short-Form Service Leadership Attitude Scale (SLA-SF-46)

The Long-Form Service Leadership Attitude Scale was developed based on the Service Leadership Model (Shek et al., 2015b, 2018f) and the leadership literature (e.g., Page and Wong, 2000; Kopelman et al., 2008). Each of the original 132 statements presents a viewpoint on the nature of leadership and how a leader ought to conduct him/herself, where participants evaluated the extent to which they concurred with each item (Shek et al., 2017). A six-point Likert scale was used (1 = strongly disagree; 6 = strongly agree). Based on findings from an unpublished, quasi-experimental validation study involving 200 students from a university in Hong Kong, a shortened version of the survey containing 73 items was formed (SLA-SF-73). The SLA-SF-73 was further refined based on Exploratory Factor Analyses and Confirmatory factor analyses by using a large-scale sample (Ma et al., 2018; Shek and Chai, 2019). The final SLA-SF-46 used in the present study possesses excellent internal consistency (α = 0.93, mean inter-item correlations = 0.27). Sample items of the SLA-SF-46 are shown in Table 3.

**Table 3:** Sample items of the Short-Form Service Leadership Attitude Scale (SLA-SF-46).

Items	Strongly Disagree	Disagree	Slightly Disagree	Slightly Agree	Agree	Strongly Agree
Sample item 1: Good leaders serve with a genuine heart.	1	2	3	4	5	6
Sample item 2: Good leaders give high priority to ethical issues.	1	2	3	4	5	6

*All sample items were slightly re-phrased to avoid practice effect.*

The present study is primarily concerned with the validation findings for the SLB-SF-65. Details in relation to the validation of the SLA-SF-73 and the SLK-SF-50 are discussed in two separate papers (Shek et al., 2018d,f).

#### External Criterion Measures

Four external criterion scales adopted from the personality and leadership literature were used to gauge the convergent validity of the SLB-SF-65. These included the Revised Servant Leadership Profile (RSLP), Moral Self-Concept Scale (MSC), Leadership Efficacy Scale (LEF), and the Interpersonal Reactivity Index (IRI).

The RSLP was developed by Wong and Page (2003) to examine servant leadership. In this study, we selected five factors of the RSLP, which included 20 items that were highly relevant to the SLAM curriculum framework (Hong Kong Institute of Service Leadership and Management Limited [HKI-SLAM], 2013). These five factors are empowering and developing others (five items), serving others (seven items), open, participatory leadership (two items), inspiring leadership (two items), and courageous leadership (four items). The RSLP demonstrated excellent reliability in the present study (α = 0.94, mean inter-item correlations = 0.46).

The MSC was developed by Cheng (2005) to measure young people’s self-appraisal on morality. The dimensions of MSC include conduct and virtues, self-control and disciplines, and altruism. All these aspects are crucial to how a service leader conducts himself/herself (Chung and Bell, 2015). The MSC presented good internal consistency in this study (α = 0.83, mean inter-item correlations = 0.44).

The LEF was developed by Murphy (1992) to examine one’s level of confidence in his/her capacity to lead effectively. The LEF showed an acceptable internal consistency metrics (α = 0.70, mean inter-item correlations = 0.24).

The IRI was developed to assess empathy (Davis, 1983). In this study, we selected 14 items from two subscales of IRI, including empathic concern (IRI-EC, seven items) and perspective taking (IRI-PT, seven items). These two subscales are closely related to the qualities of an effective service leader (Chung and Elfassy, 2016). The IRI also showed good internal consistency in the present study (α = 0.74).

### Analysis

#### Factorial Validity

Both exploratory (EFA) and confirmatory factor analysis (CFA) were involved in the validation study. While EFA provides preliminary evidence of a theoretical factorial solution (Shek et al., 2018c), CFA serves to verify the solution and validate the construct of the instrument (Besnoy et al., 2016). This two-step analytic approach has been commonly adopted to establish factorial validity of an instrument (e.g., Park, 2014; Wu and Mohi, 2015; Swami et al., 2017). SPSS version 24.0 (IBM) was utilized to administer the EFA and analyses of reliability and convergent validity. Mplus version 6.12 (Muthén and Muthén, 1998–2010) was used to perform the CFA.

As mentioned above, EFA was conducted on the SLB-SF-65 using a principal component analysis (PCA) with varimax rotation. Related findings suggested a six-factor structure of the trimmed scale (i.e., SLB-SF-48), which retained 48 items with factor loadings larger than 0.50. Besides, identical PCAs were performed on subsets A (*N* = 2,246) and B (*N* = 2,240). Tucker’s coefficients of congruence (*r*_c_) were used to evaluate the factor structure stability across the two subsets. SLB-SF-48 was revealed to be internally consistent and have a stable factorial structure. The item loadings of all 48 items ranged from 0.50 to 0.76. Details regarding the EFA and the steps involved in forming the initial 48-item behavior scale were reported in another paper (Shek et al., 2018c). The present paper primarily reports the findings of the CFA performed on the subset B (*N* = 2,240), internal consistency, convergent and factorial validity of the final version of the Service Leadership Behavior Scale (SLB-SF-38).

Before performing the main analyses, we conducted a preliminary screening to examine the skewness and kurtosis of the variables involved. Chou and Bentler’s (1995) criteria was adopted (skewness < |2|; kurtosis < |7|). Then we administered the multigroup CFA (MGCFA) to establish measurement invariance of the final model. A series of MGCFAs were conducted following the steps suggested by van de Schoot et al. (2012), which specified configural, metric, scalar and error variance invariance models to be examined. The MGCFAs were performed on three pairs of subsamples under subset B (*N* = 2,240). One pair involved males (*N* = 728) versus females (*N* = 1,498), the second pair included “odd” (*N* = 1,120) versus “even” (*N* = 1,120) groups based on case number, and the third pair included “young” (*N* = 1,120) versus “old” (*N* = 1,120) groups based on student age. Due to length constraints and the similarity of the analyses between gender and age groups, the present study mainly reported the detailed information of measurement invariance tests on the first two pairs of subsamples.

The model fit was examined by indices including the chi-square (*χ*^2^), comparative fit index (CFI), Bentler-Bonett Non-Normed fit index (NNFI), root mean square error of approximation (RMSEA), and the standardized root mean square residual (SRMR). We adopted the cutoff of 0.90 for both CFI and NNFI as indicators of adequate fit (Kline, 2005; Awang, 2012; van de Schoot et al., 2012). Regarding RMSEA and SRMR, a value below 0.80 and 0.10, respectively, should represent reasonable fit (Byrne, 1998; Hirsh, 2010). Considering that *χ*^2^ test is sensitive to sample size and model complexity, we adopted difference-in-CFI (ΔCFI) as the main invariance test indicator (Cheung and Rensvold, 2002). Particularly, as proposed by Cheung and Rensvold’s (2002), a ΔCFI below (or equal to) 0.01 suggests invariance (Schmitt and Kuljanin, 2008; Byrne, 2010). Additionally, modification indices (M.I.s) of items were reviewed upon marginal model fit. Some researchers suggested that items with extreme M.I.s (i.e., >40.0) should be dropped (Anderson and Gerbing, 1988, p. 417).

#### Reliability and Convergent Validity

Cronbach’s alpha values and mean inter-item correlations were used as the indicators of reliability of the behavior scale and the subscales derived. We also examined the convergent validity of the behavior scale in terms of its correlation with relevant constructs such as servant leadership and empathy measured by external measures (e.g., RSLP, IRI). Specifically, considering that servant leadership, moral self-concept, leadership efficacy and empathy were all key behavioral prerequisites of a service leader (see Chung and Bell, 2015; Chung and Elfassy, 2016), we hypothosized a positive and significant correlation between the service leadership behavior scale and the RSLP (Hypothesis 1), MSC (Hypothesis 2), LEF (Hypothesis 3), and IRI (Hypothesis 4), respectively.

The convergent validity of the behavior scale could be further evidenced by its correlation with the SLA-SF-46 and the SLK-SF-40. Since all three scales were constructed to examine different facets of service leadership, we predicted a positive and significant correlation between the behavior scale (and its subscales) with both the SLA-SF-46 (Hypothesis 5) and the SLK-SF-40 (Hypothesis 6).

## Results

### Data Screening and Descriptive Statistics

As detailed in Table 4, Cronbach’s alpha values and mean inter-item correlations showed good internal consistency of the initial six-factor solution (see Figure 1). No abnormal findings were found regarding each variable’s means, standard deviation, univariate skewness and kurtosis values. In short, the descriptive analyses informed the normality of data distribution, rendering the use of Maximum Likelihood (ML) estimation method appropriate. The sample size of the present study (*N* = 2,240) was also adequately powered (MacCallum et al., 1999).

**Table 4:** Descriptive statistics and Reliability indices of the original 48-item model (SLB-SF-48).

	Factors						
	Reliability statistics		Descriptive statistics			Skewness	Kurtosis
Factors	α	Mean inter-item correlations	Items	Mean	*SD*	Value	Value
(1) Self-improvement and Self-Reflections	0.930	0.527	Q47	4.56	1.00	−0.729	0.813
(12 items)			Q48	4.65	0.94	−0.719	0.995
			Q49	4.50	0.98	−0.695	0.863
			Q50	4.42	1.06	−0.690	0.504
			Q51	4.72	0.88	−0.742	1.393
			Q52	4.66	0.95	−0.703	0.892
			Q53	4.61	0.93	−0.626	0.762
			Q54	4.56	0.98	−0.756	0.959
			Q55	4.67	0.97	−0.689	0.649
			Q56	4.70	0.94	−0.792	1.161
			Q57	4.61	0.95	−0.705	0.969
			Q58	4.60	0.98	−0.586	0.524
(2) People and Principles Orientation	0.905	0.446	Q01	4.38	0.96	−0.780	1.135
(12 items)			Q32	4.55	0.95	−0.659	0.842
			Q37	4.71	0.92	−0.903	1.736
			Q38	4.79	0.90	−0.800	1.163
			Q39	4.77	0.93	−0.756	0.974
			Q40	4.48	0.99	−0.680	0.685
			Q41	4.67	0.88	−0.706	1.153
			Q42	4.65	0.88	−0.640	0.982
			Q60	4.75	0.91	−0.887	1.635
			Q61	4.58	0.93	−0.849	1.479
			Q62	4.64	0.85	−0.638	1.117
			Q65	4.82	0.86	−0.807	1.406
(3) Resilience	0.888	0.502	Q11	4.19	1.10	−0.513	−0.064
(8 items)			Q12	4.19	1.09	−0.452	−0.089
			Q13	4.31	1.04	−0.587	0.331
			Q15	4.44	0.96	−0.624	0.740
			Q16	4.50	0.96	−0.526	0.468
			Q17	4.43	0.97	−0.530	0.480
			Q18	4.27	1.09	−0.586	0.226
			Q19	4.23	1.11	−0.514	0.025
(4) Social Competence	0.898	0.556	Q20	4.65	0.92	−0.738	1.121
(7 items)			Q21	4.64	0.94	−0.896	1.347
			Q22	4.70	0.91	−0.795	1.344
			Q24	4.51	0.93	−0.751	1.138
			Q25	4.36	1.00	−0.576	0.420
			Q26	4.39	0.96	−0.585	0.436
			Q27	4.48	0.97	−0.622	0.665
(5) Problem-Solving	0.875	0.539	Q04	4.43	0.93	−0.463	0.305
(6 items)			Q05	4.22	1.00	−0.467	0.191
			Q06	4.56	0.97	−0.614	0.585
			Q07	4.53	0.95	−0.544	0.487
			Q08	4.48	0.98	−0.629	0.565
			Q09	4.38	0.95	−0.610	0.726
(6) Mentorship	0.847	0.647	Q43	4.35	0.98	−0.572	0.472
(3 items)			Q44	4.19	1.04	−0.496	0.192
			Q45	4.18	1.07	−0.540	0.146
Service Leadership Behavior Scale	0.966	0.377	–	4.51	0.60	–	–
(48 items; SLB-SF-48)							

*N = 2,240. α, Cronbach’s alpha coefficients. SD, standard deviation.*

### Factorial Validity Assessment

#### Factor Structure of the Initial Model: SLB-SF-48

Based on the original EFA solution, the findings revealed that the initial model (SLB-SF-48) fit the data reasonably well (RMSEA = 0.061; SRMR = 0.046), although some indices (CFI = 0.86; NNFI = 0.86) fell short of the recommended levels (Aquino and Reed, 2002). After reviewing the modification indices (M.I.s), we further removed 10 items reflecting double factor loadings or a strong residual covariance with other items or factors (see Table 5) (Anderson and Gerbing, 1988; Awang, 2012). The alpha values remained high when an item was removed from the scale (ranged from 0.853 to 0.925, see Table 5). The resultant six-factor, 38-item model (Model 1) was subjected to the second CFA.

**Table 5:** Items removed from SLB-SF-48 due to extreme modification indices.

Factors	Items removed	α if an item is deleted	Modification indices (M.I.s) with items within the same factor	
			Items	Modification indices
Problem-solving	Q09	0.853	Q06	42.28
			Q08	193.93
Resilience	Q11	0.880	Q12	509.64
			Q15	76.25
			Q16	45.13
			Q17	46.10
Social Competence	Q25	0.880	Q21	67.80
			Q22	101.95
			Q24	89.73
			Q26	262.31
			Q27	80.86
Social Competence	Q26	0.885	Q20	99.04
			Q21	107.36
			Q22	88.42
			Q25	262.31
			Q27	176.58
People and Principles Orientation	Q39	0.896	Q38	156.48
			Q40	51.36
People and Principles Orientation	Q41	0.895	Q42	380.63
			Q60	45.39
People and Principles Orientation	Q61	0.898	Q42	42.86
			Q60	131.43
			Q62	194.29
			Q65	50.34
Self-improvement and Self-reflection	Q47	0.925	Q48	81.84
			Q50	180.82
			Q52	69.20
			Q54	60.44
Self-improvement and Self-reflection	Q53	0.924	Q49	47.03
			Q52	227.19
			Q54	227.11
			Q57	43.85
Self-improvement and Self-reflection	Q57	0.924	Q53	43.85
			Q55	52.08
			Q56	78.19
			Q58	179.73

*Only M.I.s (with items within the same factor) larger than 40.00 were shown.*

#### Factor Structure of the Modified Model: SLB-SF-38

As detailed in Table 6, the fit indices considerably improved after the deletion of problematic items (CFI = 0.902; NNFI = 0.894; RMSEA = 0.056; SRMR = 0.045). The M.I.s of this 38-item model (Model 1) were further scrutinized. Three pairs of parameters indicated high covariance, including items Q04 and Q05 (M.I. = 239.75), Q18 and Q19 (M.I. = 150.34), and Q49 and Q50 (M.I. = 399.57).

**Table 6:** Goodness-of-fit statistics for the modified CFA models.

Model	Modifications	Comparative models	*χ*^2^	Δ*χ*^2^	*df*	Δ*df*	*p*	CFI	NNFI	RMSEA (90% CI)	SRMR
0	Original model (Six-factor SLB-SF-48)		9,939.86		1,065			0.864	0.856	0.061 (0.060 – 0.062)	0.046
1	10 items deleted (see Table 5) from Model 0		5,297.81		650			0.902	0.894	0.056 (0.055 – 0.058)	0.045
		1 versus 0		4642.05		415	<0.001				
2	Model 1 + correlated errors of Q04 and Q05		5,062.22		649			0.907	0.900	0.055 (0.054 – 0.057)	0.046
		2 versus 1		235.59		1	<0.001				
3	Model 2 + correlated errors of Q18 and Q19		4,912.93		648			0.910	0.903	0.054 (0.053 - 0.056)	0.046
		3 versus 2		149.29		1	<0.001				
4	Model 3 + correlated errors of Q49 and Q50		4,496.31		647			0.919	0.912	0.052 (0.050 – 0.053)	0.046
		4 versus 3		416.62		1	<0.001				
5	Model 4: Males (*N* = 742; separate testing)		1,896.30		647			0.922	0.915	0.051 (0.048 – 0.054)	0.043
6	Model 4: Females (*N* = 1,498; separate testing)		3,606.68		647			0.906	0.898	0.055 (0.054 – 0.057)	0.051
7	Model 4: Odd (*N* = 1,120; separate testing)		2,683.30		647			0.917	0.910	0.053 (0.051 – 0.055)	0.047
8	Model 4: Even (*N* = 1,120; separate testing)		2,837.68		647			0.906	0.898	0.055 (0.053 – 0.057)	0.050
Criterion for goodness-of-fit			–		–			≥0.90	≥0.90	<0.08	<0.10

*N_whole_ = 2,240. All χ^2^ values were statistically significant at p < 0.001 (two-tailed); Δχ^2^, change in χ^2^ compared to the previous model; Δdf, change in degrees of freedom compared to the previous model; CFI, Comparative Fit Index; RMSEA, Root Mean Square Error of Approximation; CI, Confidence Interval; NNFI, Bentler–Bonett Non-Normed Fit Index; SRMR, Standardized Root Mean Square Residual.*

Byrne (1998) contended that these extreme M.Is. may be attributed to the unique characteristics that these items shared in content. Accordingly, these three pairs of scale items were revisited. First, both items Q04 and Q05 refer to problem-solving. Second, items Q18 and Q19 measure specifically participants’ adaptive coping strategies amidst adversity. Third, both items Q49 and Q50 tap into participants’ mindset or competence in goal-setting. In a nutshell, all these observations pointed toward an overlap in content amongst the three pairs of items, which justified the inclusion of error correlations amongst these pairs (Shek and Yu, 2014). Consequently, three modified models were re-specified based on Model 1. More specifically, Model 2 included a correlation between errors of items Q04 and Q05; Model 3 built on Model 2 by incorporating an error covariance of items Q18 and Q19; Model 4 further added to Model 3 by co-varying the errors of items Q49 and Q50. Table 6 presents the goodness-of-fit statistics of Model 1 to Model 4 so as the initial six-factor 48-item solution (Model 0).

**FIGURE 2:**
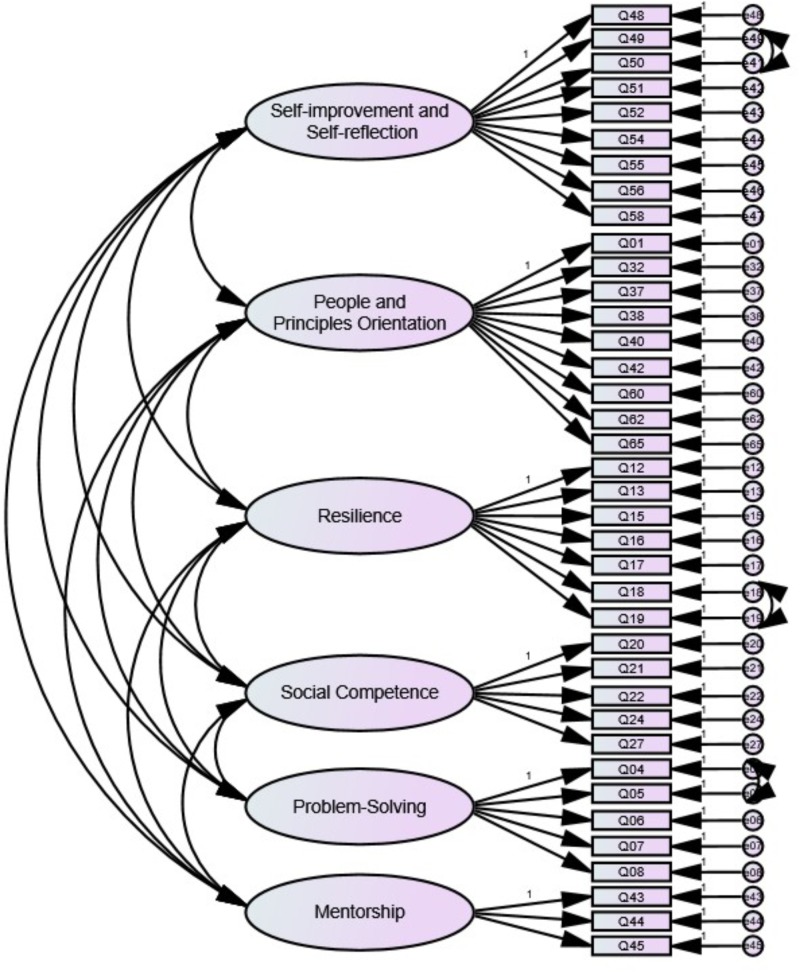
The final six-factor factorial model (Model 4; i.e., SLB-SF-38) of the Service Leadership Behavior Scale.

All indices represented the adequate fit of Model 4 to the data (*χ*^2^(647) = 4,496.31; CFI = 0.919; NNFI = 0.912, RMSEA = 0.052 [90% CI: 0.050–0.053]; SRMR = 0.046). The results of Chi-square tests showed that Model 2, Model 3 and Model 4 demonstrated significant improvement compared to Model 1, Model 2 and Model 3, respectively. We also referred to the difference-in-CFI (ΔCFI) indicator with reference to Cheung and Rensvold’s (2002) proposed cutoff of | 0.01| as the benchmark. The results showed that Model 4 significantly improved than Model 1. As a result, Model 4 was accepted as the final model (SLB-SF-38, see Figure 2).

As shown in Table 7, the standardized factor loadings of all 38 items were above 0.50 (*p* < 0.001, two-tailed), and squared multiple correlations were greater than 0.25 (*p* < 0.001, two-tailed).

**Table 7:** Standardized factor loadings for the six subscales of SLB-SF-38 (Model 4).

Subscales	Items	Factor loadings	SMC
(1) Self-improvement and Self-reflection	Q48	0.72	0.52
	Q49	0.65	0.42
	Q50	0.62	0.39
	Q51	0.76	0.58
	Q52	0.75	0.57
	Q54	0.74	0.54
	Q55	0.76	0.58
	Q56	0.80	0.65
	Q58	0.71	0.51
(2) People and Principles Orientation	Q01	0.51	0.26
	Q32	0.62	0.38
	Q37	0.71	0.51
	Q38	0.73	0.53
	Q40	0.63	0.40
	Q42	0.70	0.49
	Q60	0.69	0.47
	Q62	0.63	0.39
	Q65	0.65	0.43
(3) Resilience	Q12	0.62	0.38
	Q13	0.71	0.50
	Q15	0.77	0.59
	Q16	0.78	0.60
	Q17	0.78	0.61
	Q18	0.67	0.45
	Q19	0.66	0.44
(4) Social Competence	Q20	0.79	0.63
	Q21	0.83	0.69
	Q22	0.81	0.66
	Q24	0.72	0.51
	Q27	0.64	0.41
(5) Problem-Solving	Q04	0.65	0.42
	Q05	0.64	0.41
	Q06	0.79	0.62
	Q07	0.79	0.63
	Q08	0.74	0.55
(6) Mentorship	Q43	0.75	0.56
	Q44	0.86	0.74
	Q45	0.82	0.67

*N = 2,240. SMC, Squared multiple correlations. All standardized factor loadings (STDYX metrics) and SMC were statistically significant at p < 0.001 (two-tailed).*

#### Invariance Tests Across Genders

Model 4 was tested separately by gender in Model 5 and Model 6 to gauge its factorial stability (Byrne, 1998; Shek and Ma, 2010). As shown in Table 6, both models demonstrated adequate fit to the data in both the male (Model 5: *χ*^2^ (647) = 1,896.30; CFI = 0.922; NNFI = 0.915, RMSEA = 0.051 [90% CI: 0.048 to 0.054]; SRMR = 0.043) and female subsamples (Model 6: *χ*^2^ (647) = 3,606.68; CFI = 0.906; NNFI = 0.898, RMSEA = 0.055 [90% CI: 0.054 to 0.057]; SRMR = 0.051). As illustrated in Table 8, all factor loadings and the squared multiple correlations in the two models were significant at *p* < 0.001, two-tailed.

**Table 8:** Complete standardized factor loadings and squared multiple correlations for Model 5 to Model 8.

	Model 5 (Males; *N* = 742)		Model 6 (Males; *N* = 1,498)		Model 7 (Odd; *N* = 1,120)		Model 8 (Even; *N* = 1,120)	
	Factor loadings	SMC	Factor loadings	SMC	Factor loadings	SMC	Factor loadings	SMC
**Factor 1. Self-improvement and Self-reflection**								
Q48	0.71	0.51	0.73	0.53	0.72	0.52	0.73	0.53
Q49	0.64	0.41	0.65	0.42	0.63	0.40	0.66	0.44
Q50	0.63	0.39	0.63	0.39	0.63	0.39	0.62	0.39
Q51	0.75	0.56	0.76	0.58	0.74	0.55	0.78	0.60
Q52	0.75	0.58	0.75	0.56	0.75	0.56	0.76	0.58
Q54	0.74	0.54	0.74	0.54	0.75	0.56	0.72	0.52
Q55	0.74	0.54	0.77	0.60	0.76	0.58	0.76	0.57
Q56	0.78	0.61	0.82	0.67	0.82	0.67	0.79	0.62
Q58	0.72	0.51	0.71	0.50	0.71	0.51	0.72	0.51
**Factor 2. People and Principles Orientation**								
Q01	0.59	0.35	0.46	0.21	0.52	0.27	0.51	0.26
Q32	0.65	0.42	0.59	0.34	0.64	0.41	0.59	0.35
Q37	0.74	0.55	0.69	0.48	0.73	0.53	0.71	0.50
Q38	0.75	0.57	0.71	0.50	0.74	0.55	0.72	0.52
Q40	0.68	0.46	0.60	0.36	0.64	0.41	0.63	0.39
Q42	0.73	0.54	0.67	0.45	0.70	0.49	0.70	0.48
Q60	0.70	0.58	0.68	0.46	0.69	0.48	0.68	0.47
Q62	0.64	0.67	0.62	0.38	0.63	0.40	0.62	0.39
Q65	0.65	0.65	0.65	0.43	0.66	0.44	0.65	0.42
**Factor 3. Resilience**								
Q12	0.58	0.34	0.64	0.41	0.60	0.36	0.63	0.40
Q13	0.68	0.46	0.73	0.53	0.72	0.52	0.69	0.48
Q15	0.77	0.60	0.76	0.58	0.76	0.57	0.78	0.61
Q16	0.75	0.57	0.79	0.63	0.77	0.59	0.79	0.62
Q17	0.78	0.61	0.78	0.60	0.81	0.65	0.75	0.56
Q18	0.69	0.47	0.66	0.43	0.67	0.45	0.67	0.45
Q19	0.66	0.44	0.66	0.44	0.71	0.50	0.61	0.37
**Factor 4. Social Competence**								
Q20	0.78	0.61	0.80	0.64	0.82	0.68	0.76	0.58
Q21	0.81	0.66	0.84	0.71	0.83	0.70	0.83	0.69
Q22	0.80	0.63	0.81	0.66	0.81	0.66	0.81	0.65
Q24	0.72	0.52	0.71	0.50	0.74	0.54	0.69	0.48
Q27	0.68	0.46	0.61	0.37	0.67	0.45	0.61	0.37
**Factor 5. Problem-Solving**								
Q04	0.65	0.42	0.65	0.43	0.66	0.44	0.64	0.41
Q05	0.67	0.45	0.63	0.39	0.64	0.41	0.65	0.42
Q06	0.78	0.61	0.79	0.63	0.76	0.58	0.82	0.66
Q07	0.78	0.62	0.80	0.64	0.77	0.59	0.81	0.66
Q08	0.73	0.54	0.75	0.56	0.75	0.56	0.74	0.55
**Factor 6. Mentorship**								
Q43	0.76	0.58	0.74	0.55	0.76	0.57	0.74	0.55
Q44	0.82	0.67	0.89	0.79	0.85	0.73	0.87	0.76
Q45	0.80	0.65	0.82	0.68	0.81	0.66	0.82	0.67

*N_whole_ = 2,240. SMC, Squared multiple correlations. All standardized factor loadings (STDYX metrics) and SMC were statistically significant at p < 0.001 (two-tailed).*

As abovementioned, the invariance models were tested by the configural invariance model (Model 9), the metric invariance model (Model 10), the scalar invariance model (Model 11), and the error variance invariance model (Model 12). Table 9 showed the results of the Chi-square tests, which revealed no significant difference between Model 9 and 10 (Δ*χ*^2^ = 39.03, Δ*df* = 32, *p* > 0.05), but significant differences between Model 10 and 11 (Δ*χ*^2^ = 187.30, Δ*df* = 38, *p* < 0.001), and between Model 11 and 12 (Δ*χ*^2^ = 499.81, Δ*df* = 41, *p* < 0.001). As mentioned earlier, we followed Cheung and Rensvold’s suggestion (Cheung and Rensvold, 2002) and referred to the value of Δ CFI.

**Table 9:** Summary of goodness-of-fit for invariance tests: multigroup comparisons.

Model description	Comparative model	*χ*^2^	Δ*χ*^2^	*df*	Δ*df*	Statistical significance	CFI	ΔCFI	NNFI	ΔCFI ≤ |0.01|?	RMSEA (90% CI)
**Gender invariance**											
(9) Configural invariance	–	5,502.98	–	1,294	–	–	0.912	–	0.904	–	0.054 (0.052–0.055)
(10) Metric invariance	–	5,542.01	–	1,326	–	–	0.912	–	0.906	–	0.053 (0.052–0.055)
	10 versus 9	–	39.03	–	32	N.S.	–	<0.001	–	Yes	–
(11) Scalar invariance	–	5,729.31	–	1,364	–	–	0.908	–	0.906	–	0.053 (0.052–0.055)
	11 versus 10	–	187.30	–	38	*p* < 0.001	–	0.004	–	Yes	–
(12) Error variance invariance	–	6,229.12	–	1,405	–	–	0.899	–	0.899	–	0.055 (0.054–0.057)
	12 versus 11	–	499.81	–	41	*p* < 0.001	–	0.009	–	Yes	–
**Subgroup invariance**											
(13) Configural invariance	–	5,520.98	–	1,294	–	–	0.912	–	0.904	–	0.054 (0.053–0.055)
(14) Metric invariance	–	5,567.64	–	1,326	–	–	0.912	–	0.906	–	0.053 (0.052–0.055)
	14 versus 13	–	46.66	–	32	*p* < 0.05	–	<0.001	–	Yes	–
(15) Scalar invariance	–	5,608.20	–	1,364	–	–	0.912	–	0.909	–	0.053 (0.051–0.054)
	15 versus 14	–	40.56	–	38	N.S.	–	<0.001	–	Yes	–
(16) Error variance invariance	–	5,710.42	–	1,405	–	–	0.910	–	0.910	–	0.052 (0.051–0.054)
	16 versus 15	–	102.22	–	41	*p* < 0.001	–	0.002	–	Yes	–

*N_whole_ = 2,240; N_males_ = 742; N_females_ = 1,498; N_odd_ = 1,120; N_even_ = 1,120; CFI, Comparative Fit Index; RMSEA, Root Mean Square Error of Approximation; CI, confidence interval; Δχ^2^, change in χ^2^ compared to the previous model; Δdf, change in degrees of freedom compared to the previous model; N.S., Δχ^2^ not significant at p < 0.05; ΔCFI, change in CFI compared to the previous model; ΔCFI ≤ |0.01|?, Was the change in CFI not larger than the |0.01|-cutoff?; Model 9 and Model 13, no equality constraints were imposed; Model 10 and Model 14, equality constraints were imposed on all factor loadings; Model 11 and Model 15, equality constraints were imposed on all factor loadings and intercepts of the measured variables; Model 12 and Model 16, equality constraints were imposed on all factor loadings, intercepts, and residual variances.*

As shown in Table 9, Model 9 in which no quality constraint was postulated fit adequately with the data (*χ*^2^ (1,294) = 5,502.998; CFI = 0.912; NNFI = 0.904, RMSEA = 0.054 [90% CI: 0.052 to 0.055]; SRMR = 0.049), suggesting invariance of the overall factorial structure across genders. In Model 10, factor loadings were constrained to be equal across genders. The value of ΔCFI (<0.001) compared to Model 9 was below Cheung and Rensvold’s (2002) proposed cutoff (0.01), suggesting invariance in factor loadings as well across genders.

In Model 11, equality constraints were placed upon both factor loadings and measurement intercepts across the male and female groups. The value of ΔCFI (0.004) denoted invariance in measurement intercepts of each item across genders (see Table 9).

Lastly, in Model 12 we constrained the error variance, factor loading, and measurement intercept of each variable to be equal across genders to establish error variance invariance model (Model 12). The value of ΔCFI (0.009, see Table 9) was again below 0.01, suggesting that same level of measurement error was present for each item between males and females (Milfont and Fischer, 2010, p. 115).

#### Invariance Tests Across Other Subsamples

Following Shek and colleagues’ procedure (Shek and Ma, 2010, 2014; Shek and Yu, 2014), subset B (*N* = 2,240) was further divided into group “odd” (*N* = 1,120) and group “even” (*N* = 1,120) based on case number. Both groups were subjected to the identical set of invariance tests as reported above. As shown in Table 6, Model 4 fitted reasonably well with the dataset in both the odd (Model 7: *χ*^2^ (647) = 2,683.30; CFI = 0.917; NNFI = 0.910, RMSEA = 0.053 [90% CI: 0.051 to 0.055]; SRMR = 0.047) and even groups (Model 8: *χ*^2^ (647) = 2,837.68; CFI = 0.906; NNFI = 0.898, RMSEA = 0.055 [90% CI: 0.053–0.057]; SRMR = 0.050). These findings provided basis for the ensuing series of MGCFAs, which served to establish measurement invariance across the two subsamples.

In Model 13, no equality constraints were imposed. As illustrated in Table 9, the goodness-of-fit indices of Model 13 exhibited acceptable fit to the data (*χ*^2^(1,294) = 5,520.98; CFI = 0.912; NNFI = 0.904, RMSEA = 0.054 [90% CI: 0.053 to 0.055]; SRMR = 0.048), suggesting configural invariance. We further constrained the factor loadings to be equal in Model 14 and compared it with the baseline Model 13. The result of *χ*^2^ test was significant at the 0.05 level (Δ*χ*^2^ = 46.66, Δ*df* = 32, *p* < 0.05). The resultant value of ΔCFI (<0.001) provided support for the metric invariance across the two subsamples. In Model 15, equality constraints were further placed on the measurement intercepts of all items. The *χ*^2^ test showed a non-significant result (Δ*χ*^2^ = 40.56, Δ*df* = 38, *p* > 0.05). Likewise, the value of ΔCFI (<0.001) derived from the comparison between Model 14 and Model 15 conveyed scalar invariance. In Model 16 the error variance, factor loading and measurement intercept were held equal for every item across both subsamples. Although the *χ*^2^ test showed a significant difference between Model 16 and 15 (Δ*χ*^2^ = 76.56, Δ*df* = 41, *p* < 0.001), the resultant value of ΔCFI (0.002) remained trivial by Cheung and Rensvold’s (2002) standard, signaling error variance invariance of the final factorial solution (SLB-SF-38) as displayed in Figure 2.

Besides, we also examined the measurement invariance across age groups by dividing subset B (*N* = 2,240) into two groups based on student age. The “Young” Group (*N* = 1,120, mean age = 19.17 years, *SD* = 0.76) and “Old” Group (*N* = 1,120, mean age = 21.71, *SD* = 1.24) were subjected to the same invariance tests mentioned above. Same as gender invariance, the resultant values of ΔCFI (≤0.01) also supported configural, metric, scarlar and error variance invariance of the factorial structure between the two age groups.

In summary, the present findings provided strong support for the factorial validity of the 38-item Service Leadership Behavior Scale (SLB-SF-38). Apart from exhibiting adequate fit to the data, the strong factorial stability of the SLB-SF-38 was underscored by the series of invariance test performed based on groups defined by gender and age as well as with randomly assigned subjects. Specifically, measurement invariance of the SLB-SF-38 was supported in terms of configural, metric, scalar, and error variance invariance.

### Reliability of the Measures

As indicated in Table 10, the SLB-SF-38 showed excellent reliability (α = 0.96, mean inter-item correlations = 0.38). All its six subscales also demonstrated good to excellent reliability in the present study (αs > 0.84, mean inter-item correlations > 0.35). The inter-correlations among the SLB-SF-38 and the subscales ranged from 0.42 to 0.87 (*p* < 0.001, two-tailed). These findings underscored the strong internal consistency of the SLB-SF-38 and the subscales.

**Table 10:** Correlation coefficients, mean inter-item correlations and Cronbach’s alpha amongst the six subscales and the whole scale.

		Subscales					
Subscales	Cronbach’s alpha (Mean inter-item correlations)	1	2	3	4	5	6
(1) Self-improvement and Self-reflection	0.909 (0.530)	–	–	–	–	–	–
(2) People and Principles Orientation	0.868 (0.426)	0.70	–	–	–	–	–
(3) Resilience	0.880 (0.515)	0.61	0.57	–	–	–	–
(4) Social Competence	0.868 (0.570)	0.61	0.69	0.64	–	–	–
(5) Problem-Solving	0.853 (0.538)	0.58	0.50	0.61	0.52	–	–
(6) Mentorship	0.847 (0.647)	0.58	0.58	0.50	0.49	0.42	–
38-item Service Leadership Behavior Scale	0.958 (0.377)	0.87	0.85	0.82	0.81	0.74	0.70

*N = 2,240. All correlation coefficients are statistically significant at p < 0.001 (two-tailed).*

### Convergent Validity Assessment

#### Correlation With External Criterion Measures

As shown in Table 11, consistent with Hypotheses 1 to 4, correlational findings revealed the significant (*p* < 0.001, two-tailed) and positive association between the SLB-SF-38 (inclusive of all subscales) and the RSLP (*r*s ranging from 0.49 to 0.79), MSC (*r*s ranging from 0.37 to 0.66), LEF (*r*s ranging from 0.37 to 0.52) and IRI (*r*s ranging from 0.20 to 0.55). These findings provided convergent evidence for the validity of the SLB-SF-38, given that this scale was moderately related to several constructs outlining the behavioral characteristics of a service leader (Chung and Elfassy, 2016).

**Table 11:** Correlations with external criterion scales (and subscales).

	External criterion scales (and subscales)					
	RSLP	MSC	LEF	IRI	IRI-EC	IRI-PT
38-item Service Leadership Behavior Scale	0.79	0.66	0.52	0.44	0.31	0.47
Subscale 1: Self-improvement and Self-reflection	0.69	0.58	0.45	0.38	0.28	0.40
Subscale 2: People and Principles Orientation	0.78	0.70	0.37	0.55	0.44	0.53
Subscale 3: Resilience	0.57	0.44	0.42	0.23	0.12	0.30
Subscale 4: Social Competence	0.62	0.58	0.46	0.42	0.32	0.43
Subscale 5: Problem-Solving	0.49	0.37	0.45	0.20	0.10	0.26
Subscale 6: Mentorship	0.64	0.45	0.40	0.25	0.16	0.29

*N = 2,240. All correlation coefficients are statistically significant at p < 0.001 (two-tailed). RSLP, Revised Servant Leadership Profile; MSC, Moral Self-Concept; LEF, Leadership Efficacy; IRI, Interpersonal Reactivity Index; IRI-EC, Subscale “Empathic Concern”; IRI: PT, Subscale “Perspective Taking.”*

#### Correlation With Other Service Leadership Measures

Furthermore, findings of correlational analyses between the SLB-SF-38 and the final versions of the Service Leadership Attitude (SLA-SF-46) and Knowledge (SLK-SF-40) Scales are summarized in Table 12. Discussions in relation to the validation of the eight-factor SLA-SF-46 as well as the one-factor SLK-SF-40 are featured in two other papers. The SLB-SF-38 was overall moderately and positively linked to the SLA-SF-46 (*r* = 0.58) and also positively linked to the SLK-SF-40 (*r* = 0.19). The subscales of the SLB-SF-38 were also correlated positively and significantly with both the SLA-SF-46 and the SLK-SF-40. Although some occasional non-significant and unexpected results were observed, the results of correlational analyses supported Hypotheses 5 and 6.

**Table 12:** Correlations with other Service Leadership scales (and subscales) under validation.

	SLK-SF-40	SLA-F1	SLA-F2	SLA-F3	SLA-F4	SLA-F5	SLA-F6	SLA-F7	SLA-F8	SLA-SF-46
38-item Service Leadership Behavior Scale	0.19	0.51	0.49	0.51	0.40	0.50	0.47	0.28	−0.05	0.58
Subscale 1: Self-improvement and Self-reflection	0.20	0.46	0.42	0.42	0.33	0.44	0.43	0.20	−0.03^n.s.^	0.50
Subscale 2: People and Principles Orientation	0.28	0.52	0.55	0.52	0.41	0.56	0.47	0.28	0.02^n.s.^	0.62
Subscale 3: Resilience	0.06	0.35	0.33	0.37	0.29	0.32	0.33	0.23	−0.09	0.39
Subscale 4: Social Competence	0.20	0.42	0.43	0.41	0.31	0.44	0.38	0.23	0.02^n.s.^	0.49
Subscale 5: Problem-Solving	0.12	0.38	0.34	0.34	0.25	0.33	0.36	0.13	−0.05	0.39
Subscale 6: Mentorship	−0.08	0.26	0.26	0.40	0.35	0.22	0.22	0.27	−0.18	0.32

*N = 2,240. Unless otherwise specified by superscript ”n.s.” which denotes statistical non-significance, all correlation coefficients are significant at *p* < 0.05 (two-tailed). SLK-SF-40, Scale score of the one-factor, 40-item Service Leadership Knowledge Scale; SLA-SF-46, Scale score of the eight-factor, 46-item Service Leadership Attitude Scale; SLA-F1, Factor ”Vision and competence”; SLA-F2, Factor ”People orientation”; SLA-F3, Factor ”Caring disposition”; SLA-F4, Factor ”Ethical role model”; SLA-F5, Factor ”Social competence”; SLA-F6, Factor ”Self-understanding and reflection”; SLA-F7, Factor ”Positive view about human beings”; SLA-F8, Factor 8 ”Unchangeable and dark human nature.”*

To conclude, the present findings offered solid and consistent evidence for the construct validity of the SLB-SF-38. The main scale and the six subscales were correlated with a series of well-validated measures developed to examine constructs related to service leadership. Besides, the SLB-SF-38 and the subscales were also correlated with Service Leadership Attitude Scale and Service Leadership Knowledge Scale, which assessed the different dimensions of the same underlying construct. Thus, the SLB-SF-38 is shown to be a valid and reliable measurement tool of the behavioral characteristics of a service leader.

## Discussion

The present study attempted to examine the reliability, convergent validity and dimensionality of the Short-Form Service Leadership Behavior Scale (SLB-SF-65) based on a large sample of Hong Kong undergraduates. The findings suggested the retention of 38 items, which can be grouped under six dimensions including “Self-improvement and Self-reflection,” “People and Principles Orientation,” “Resilience,” “Social Competence,” “Problem-Solving,” and “Mentorship.” The results of multi-group CFA supported the stability of this factorial structure. Both the SLB-SF-38 and the six subscales presented good internal consistency and robust convergent validity. In short, this study validated the SLB-SF-38 as a sound assessment tool to evaluate the behavioral attributes of service leaders.

There are several strengths of the present study. First, the development of the scales were driven by the Service Leadership Model, which has been extensively covered in the literature and shown to be beneficial to university students in Hong Kong (Shek and Chung, 2015; Shek et al., 2017). Second, the present study employed a large sample which accounted for 5.36% of the total 84,388 Hong Kong undergraduates in the 2016/17 academic year (University Grants Committee [UGC], 2017). This large sample contributed to the robust findings (Biau et al., 2008). Third, the present study constructed an objective and psychometrically sound measurement tool to the leadership and youth development literature. Fourth, this study validated an objective measurement assessing service leadership behaviors in a Chinese context with an important role in the global service economy.

The present six dimensions aligned well with the Service Leadership Model. First, the factor “Self-improvement and Self-reflection” (nine items) emphasizes the importance of reviewing and improving one’s own leadership behavior as a continuous quest (Chung and Bell, 2015, p. 59). The second factor “People and Principles Orientation” (9 items) is concerned with having a set of personal code of ethics and treating others with care (Chung and Elfassy, 2016). This dimension is consistent with the morality, trust, fairness and respect emphasized in Service Leadership Model. Third, the dimension “Resilience” (seven items) measures an individual’s ability to effectively respond toward stress, difficulty, and other unpleasant events in life (Shek and Lin, 2015c). This dimension can be conceptualized as an intrapersonal competence that enhances leadership effectiveness (Patel, 2012; Hatler and Sturgeon, 2013). Therefore, resilience constitutes an essential behavioral attribute of an effective service leader, and it is definitely a key component of service leadership education (Shek and Leung, 2015). The fourth factor “Social Competence” (five items) covers three aspects on one’s capacity to effectively handle social interactions. These aspects include the ability to get along with other people, to build and accordingly maintain close relationships, and to behave appropriately in social settings (see Orpinas, 2010). This factor echoes the interpersonal competence outlined in Service Leadership Model. Fifth, the dimension “Problem-Solving” (five items) measures people’s critical thinking when tackling difficult or complex issues (Altun, 2003). Problem-Solving falls into the category of intrapersonal competence as part of the service leadership education curriculum (Shek and Leung, 2015). Effective problem-solving is vital to leadership success (Mumford et al., 2000), and closely related to other intrapersonal competence such as emotion management (Mehrdad et al., 2011). Furthermore, service leaders may need to solve potentially conflicting needs of self, others, and the systems without compromising on morality. In this situation, critical thinking will help service leaders to see bigger picture and handle the problem in a timely manner (Jasovsky and Kamienski, 2007). Thus, the factor “Problem-Solving” underlies a dimension of behavioral attributes of service leadership. Lastly, the subscale “Mentorship” (three items) measures participants’ capability and willingness to support other’s development (Shek and Lin, 2015d), echoing the *Competence* and *Care* components highlighted in the Service Leadership Model. In short, the findings provide support for the “3-Cs” (*Competence*, *Character* and *Care*) of the Service Leadership Model. The results also echo the belief that both “being” (i.e., *Character* and *Care*) and “doing” (i.e., *Competence*) are important for effective leadership. The findings are pioneering in terms of constructing a validated measures of service leadership in Chinese societies.

The present study provides support for the developed tool on service leadership behavior. The findings enable cross-institutional analyses on curriculum effectiveness, and also offer robust empirical support for the Service Leadership Model (Shek and Chung, 2015; Shek et al., 2017). Theoretically speaking, the finings underscore the importance of the different dimensions of the measure as components of service leadership. This contributes to the development of the theory of service leadership.

The present study has several practical implications. First, the SLB-SF-38 can be employed to assess the impact of a service leadership training program. As students are expected to demonstrate an improvement in behavioral attributes of service leadership after completing the program, educators can use this tool to assess the change. Second, the dimensionality of the SLB-SF-38 can be used to refine service leadership education curriculum. Specifically, the curriculum materials for future service leadership training may be tuned to focus on the six dimensions identified. Third, the SLB-SF-38 can be used by employers looking for candidates possessing key behavioral attributes of an effective service leader. Finally, the developed tool can help researchers to conduct studies on service leadership in the changing service economy in the global context.

While the present study is pioneer in the area of service leadership, there are several limitations of the study. First, only undergraduate students in Hong Kong were recruited in the present study. Hence, it would be helpful to understand the psychometric properties of the measure in other student populations. Besides, to further endorse the factorial validity of the SLB-SF-38, follow-up validation studies using a sample of executives (e.g., Acar and Zehir, 2009) or managers (e.g., Yukl et al., 2008) are suggested.

Second, given that the present survey comprised over 250 items, response burden may influence the response quality (Lavrakas, 2008). Besides, content overlap could also be a “turn-off” for the respondents (Rolstad et al., 2011). In addition, although findings provide strong support for the internal consistency of the SLB, the test-retest reliability analyses can be conducted to examine the temporal stability of the measure in future. Nevertheless, our results showed good internal consistency of both the scale and the subscales (see Table 4), implying the quality responses from the participants (Oltedal et al., 2007).

Third, the SLB-SF-38 relies on participants’ self-rated leadership behavior, which may cause social desirability bias in responses. Participants may tend to provide favorable instead of truthful responses. Although we assured the participants that the responses would be kept confidential and anonymous, this limitation should be taken into account. In future, additional information collected from other informants (e.g., followers) would give a more comprehensive picture about service leadership behavior seen from different perspectives.

Finally, one can criticize that because the data are ordinal data, it is not appropriate to use parametric factor analysis. While we acknowledge this weakness of the present paper, we would like to make several arguments supporting the approach adopted in this study. Primarily, although there are contrary views, it is a common practice to treat ordinal data with several response categories as continuous data (Muthén and Kaplan, 1985). Second, it is also a common practice to apply CFA with ML estimation to test the model of Likert scale measurement (Byrne, 2010). For example, similar papers using CFA to analyze Likert scale data have been reported in some prestigious journals, including *Frontiers in Psychology* and *Psychological Assessment* (Young and Beaujean, 2011; Coates et al., 2016; Jorge-Monteiro and Ornelas, 2016; Ghislieri et al., 2017).

Third, Carifio and Perla discussed some common misunderstandings about Likert scales and regarded the claim that “because Likert scales are ordinal-level scales, only non-parametric statistical tests should be used with them” (Carifio and Perla, 2007, p. 114) as a common myth. They further pointed out that “if one is using a 5–7 point Likert response format, and particularly so for items that resemble a Likert-like scale and factorially hold together as a scale or subscale reasonably well, then it is perfectly acceptable and correct to analyze the results at the (measurement) scale level using parametric analyses techniques such as the F-Ratio or the Pearson correlation coefficients or its extensions (i.e., multiple regression and so on), and the results of these analyses should and will be interpretable as well” (Carifio and Perla, 2007, p. 115).

Fourth, we understand that other estimators (e.g., WLSMV) can be superior to ML when there are few ordinal categories. However, there are views supporting the application of ML for categorical data under specific conditions (Byrne, 2010). Some researchers have compared ML and other estimators applied for CFA analysis with ordered categorical data, such as WLSMV (Beauducel and Herzberg, 2006), WLS (Lei, 2009), GLS (Muthén and Kaplan, 1985; Hu and Bentler, 1998), and cat-LS (Rhemtulla et al., 2012). Most of these comparisons concluded that ML performed as good as or even better than other methods when (a) the data approximated a normal distribution (have mildly to moderately skewed/kurtosis variables), (b) there were more than five response categories, and (c) the sample size was not small. In this study, these three conditions were fully met. On the other hand, some researchers have highlighted the disadvantages of WLSMV. For example, Li pointed out the weaknesses of inter factor correlations and standard errors in WLSMV estimation “when the sample size is small, and/or when a latent distribution is moderately non-normal” (Li, 2016, p. 948). In addition, DiStefano and Morgan (2014) also noticed that WLSMV may produce factor correlation estimates with overestimation when dealing with five or more ordered categories.

Finally, as suggested by Rhemtulla et al. (2012), the choice of available methods should rely on data characters (e.g., sample size, model size, the normality of distribution), the characters of constructs underlying (e.g., the distribution of the constructs), and researchers’ own interests. In the present study, the data in general showed a normal distribution, the sample size was relatively large, and six response categories were used. In this regard, ML seems appropriate. As suggested by Allison et al. (1993, p. 92) recommended researchers “should consider staying with traditional parametric tests” when the above conditions are met. Obviously, ML provides better robust standard errors for factor correlations and the desirable asymptotic properties such as asymptotically efficiency (Lei, 2009; Rhemtulla et al., 2012).

In short, we understand the reviewer’s concern. We acknowledge the related limitations of the study and we suggest a future study to be conducted to provide an additional picture. Despite this limitation, the present study provides pioneer and exciting support for a pioneer scale on service leadership behavior in a Chinese context.

## Conclusion

Despite the above limitations, the present study provides evidence for a reliable and valid assessment tool of service leadership behavior. The present analyses provide a strong evidence base for the psychometric properties of the SLB-SF-38 by using a large sample of Chinese undergraduates. The current study fills the gap in the scientific literature on leadership assessment of leadership training amongst Chinese college students, and also provides practical implications for future service leadership education and research.

## Data Availability

The datasets generated for this study are available on request to the corresponding author.

## Ethics Statement

This study was approved by the Human Subjects Ethics Sub-committee (HSESC) (or its Delegate) of The Hong Kong Polytechnic University. All subjects have given written informed consent before start of the study.

## Author Contributions

DS designed the research project and contributed to all the steps of the work. DD contributed to the development of the article and revised the manuscript based on the critical comments and editing provided by DS. LM contributed to the initial data analyses and development of a rough draft of the manuscript.

## Conflict of Interest Statement

The authors declare that the research was conducted in the absence of any commercial or financial relationships that could be construed as a potential conflict of interest.

## References

[B1] Acar A. Z. Zehir C. (2009). Development and validation of a multidimensional business capabilities measurement instrument. *J. Transnatl. Manag.* 14 215–240. 10.1080/15475770903127050

[B2] Allison D. B. Gorman B. S. Primavera L. H. (1993). Some of the most common questions asked of statistical consultants: our favorite responses and recommended readings. *J. Group Psychother. Psychodrama Sociom.* 46 83–109.

[B3] Altun I. (2003). The perceived problem-solving ability and values of student nurses and midwives. *Nurse Educ. Today* 23 575–584. 10.1016/s0260-6917(03)00096-0 14554111

[B4] Anderson J. C. Gerbing D. W. (1988). Structural equation modeling in practice: a review and recommended two-step approach. *Psychol. Bull.* 103 411–423.

[B5] Aquino K. Reed A. (2002). The self-importance of moral identity. *J. Personal. Soc. Psychol.* 83 1423–1440. 12500822 10.1037//0022-3514.83.6.1423

[B6] Avolio B. J. Bass B. M. Jung D. I. (1999). Re-examining the components of transformational and transactional leadership using the multifactor Leadership. *J. Occup. Organ. Psychol.* 72 441–462. 10.1348/096317999166789

[B7] Awang Z. (2012). *A Handbook on SEM: Structural Equation Modelling Using AMOS Graphics*, 2nd Edn. Malaysia: Universiti Sultan Zainal Abidin press.

[B8] Bacon C. Benton D. Gruneberg M. M. (1979). Employers’ opinions of university and polytechnic graduates. *Vocat. Aspect Educ.* 31 95–102. 10.1080/10408347308001251

[B9] Bass B. M. (1990). From transactional to transformational leadership: learning to share the vision. *Organ. Dyn.* 18 19–31. 10.1016/0090-2616(90)90061-s

[B10] Beauducel A. Herzberg P. Y. (2006). On the performance of maximum likelihood versus means and variance adjusted weighted least squares estimation in CFA. *Struct. Equ. Mod.* 13 186–203. 10.1207/s15328007sem1302_2

[B11] Besnoy K. D. Dantzler J. Besnoy L. R. Byrne C. (2016). Using exploratory and confirmatory factor analysis to measure construct validity of the traits, aptitudes, and Behaviors Scale (TABS). *J. Educ. Gift.* 39 3–22. 10.1177/0162353215624160

[B12] Biau D. J. Kerneis S. Porcher R. (2008). Statistics in brief: the importance of sample size in the planning and interpretation of medical research. *Clin. Orthop. and Relat. Res.* 466 2282–2288. 10.1007/s11999-008-0346-9 18566874 PMC2493004

[B13] Brown M. E. Treviño L. K. (2006). Ethical leadership: a review and future directions. *Leadersh. Q.* 17 595–616. 10.1016/j.leaqua.2006.10.004

[B14] Bryson J. R. Daniels P. W. (2015). *Handbook of Service Business Management, Marketing, Innovation and Internationalisation*, Eds. Edn. Cheltenham: Edward Elgar Publishing.

[B15] Byrne B. M. (1998). *Structural Equation Modeling with LISREL, PRELIS, and SIMPLIS: Basic Concepts, Applications, and Programming.* Mahwah, NJ: Lawrence Erlbaum Associates.

[B16] Byrne B. M. (2010). *Structural Equation Modeling with AMOS: Basic Concepts, Applications, and Programming*, 2nd Edn. New York, NY: Routledge.

[B17] Carifio J. Perla R. J. (2007). Ten common misunderstandings, misconceptions, persistent myths and urban legends about Likert scales and Likert response formats and their antidotes. *J. Soc. Sci.* 3 106–116. 10.1016/0006-8993(93)90283-S

[B18] Cheng C. H. K. (2005). *The Chinese Adolescent Self-Esteem Scales (CASES): A User Manual.* Hong Kong: City University of Hong Kong Press.

[B19] Cheung G. W. Rensvold R. B. (2002). Evaluating goodness-of-fit indexes for testing measurement invariance. *Struct. Equ. Mod.* 9 233–255. 10.1097/NNR.0b013e3182544750 22551991 PMC3361901

[B20] Chou C.-P. Bentler P. M. (1995). “Estimates and tests in structural equation modeling,” in *Structural equation modeling: Concepts, Issues and Applications*, ed. Hoyle R. H. (Thousand Oaks, CA: Sage), 37–55.

[B21] Chung P. P. Y. (2015). “Where there is no vision, the people will perish,” in *Promoting Service Leadership Qualities In university Students: The case of Hong Kong* (pp. xv-xviii), eds Shek D. T. L. Chung P. P. Y. (Singapore: Springer).

[B22] Chung P. P. Y. Bell A. H. (2015). *25 Principles of Service Leadership*, 1st Edn. New York, NY: Lexingford Publishing.

[B23] Chung P. P. Y. Elfassy R. (2016). *The 12 Dimensions of a Service Leader*, 1st Edn. New York, NY: Lexingford Publishing.

[B24] Coates R. Ayers S. de Visser R. (2016). Factor structure of the edinburgh postnatal depression scale in a population-based sample. *Psychol. Assess.* 29 1016–1027. 10.1037/pas0000397 27736124

[B25] Davis M. H. (1983). Measuring individual-differences in empathy: evidence for a multidimensional approach. *J. Personal. Soc. Psychol.* 44 113–126. 10.1037//0022-3514.44.1.113

[B26] DiStefano C. Morgan G. B. (2014). A comparison of diagonal weighted least squares robust rstimation techniques for ordinal data. *Struct. Equ. Mod.* 21 425–438. 10.1080/10705511.2014.915373

[B27] Ghislieri C. Emanuel F. Molino M. Cortese C. G. Colombo L. (2017). New technologies smart, or harm work-family boundaries management? Gender differences in conflict and enrichment using the JD-R theory. *Front. Psychol.* 8:1070. 10.3389/fpsyg.2017.01070 28713300 PMC5492914

[B28] Greenleaf R. K. (1970). *The Servant as a Leader.* Indianapolis, IN: Greenleaf Center.

[B29] Greenleaf R. K. (1977). *Servant Leadership: A Journey into the Nature of Legitimate Power and Greatness.* Mahwah, NJ: Paulist Press.

[B30] Hatler C. Sturgeon P. (2013). Resilience building: a necessary leadership competence. *Nurse Lead.* 11 32–39. 10.1016/j.mnl.2013.05.007

[B31] Hirsh J. B. (2010). Personality and environmental concern. *J. Environ. Psychol.* 30 245–248. 10.1016/j.jenvp.2010.01.004

[B32] Ho J. Nesbit P. L. (2009). A refinement and extension of the self-leadership scale for the Chinese context. *J. Manag. Psychol.* 24 450–476. 10.1108/02683940910959771

[B33] Hong Y. Liao H. Hu J. Jiang K. (2013). Missing link in the service profit chain: a meta-analytic review of the antecedents, consequences, and moderators of service climate. *J. Appl. Psychol.* 98 237–267. 10.1037/a0031666 23458337

[B34] Hong Kong Institute of Service Leadership and Management Limited [HKI-SLAM] (2013). *An Overview of HKI-SLAM’s Curriculum Framework Prepared for Li & Fung’s Service Leadership Initiative*. Available at: http://hki-slam.org/files/press/An%20Overview%20of%20SLAM%20Curriculum%20Framework%20130531.pdf (accessed August 20, 2017).

[B35] Hu L. Bentler P. M. (1998). Fit indices in covariance structure modeling: sensitivity to underparameterized model misspecification. *Psychol. Methods* 3 424–453. 10.1037/1082-989X.3.4.424

[B36] Jasovsky D. A. Kamienski M. (2007). “Enhancing your critical thinking, decision making, and problem solving,” in *Nursing Leadership and Management: Theories, processes and practice* (1st Edn, ed. Jones R. P. (Philadelphia, PA: FA Davis), 151–165.

[B37] Jiang K. Chuang C.-H. Chiao Y.-C. (2015). Developing collective customer knowledge and service climate: the interaction between service-oriented high-performance work systems and service leadership. *J. Appl. Psychol.* 100 1089–1106. 10.1037/apl0000005 25486260

[B38] Jiang K. Hu J. Hong Y. Liao H. Liu S. (2016). Do it well and do it right: the impact of service climate and ethical climate on business performance and the boundary conditions. *J. Appl. Psychol.* 101 1553–1568. 10.1037/apl0000138 27504653

[B39] Jorge-Monteiro M. F. Ornelas J. H. (2016). Recovery assessment scale: testing validity with portuguese community-based mental health organization users. *Psychol. Assess.* 28 1–11. 10.1037/pas0000176 26121387

[B40] Kline R. B. (2005). *Principles and Practice of Structural Equation Modeling*, 2nd Edn. New York: The Guilford Press.

[B41] Kopelman R. E. Prottas D. J. Davis A. L. (2008). Douglas McGregor’s theory X and Y: toward a construct-valid measure. *J. Manag. Issues* 20 255–271.

[B42] Lavrakas P. J. (2008). *Encyclopedia of Survey Research Methods.* Thousand Oaks, CA: SAGE.

[B43] Lei P. W. (2009). Evaluating estimation methods for ordinal data in structural equation modeling. *Q. Q.* 43 495–507. 10.1007/s11135-007-9133-z 30684226

[B44] Li C. H. (2016). Confirmatory factor analysis with ordinal data: comparing robust maximum likelihood and diagonally weighted least squares. *Behav. Res. Methods* 48 936–949. 10.3758/s13428-015-0619-7 26174714

[B45] Lytle R. S. Hom P. W. Mokwa M. P. (1998). SERV^∗^OR: a managerial measure of organizational service-orientation. *J. Retailing* 74 455–489. 10.1016/s0022-4359(99)80104-3

[B46] Ma C. M. Shek D. T. Chandra Y. (2018). Development of the attitude to service leadership scale in hong kong [Special issue]. *Int. J. Child Adolesc. Health* 11 405–414.

[B47] MacCallum R. C. Widaman K. F. Zhang S. Hong S. (1999). Sample size in factor analysis. *Psychol. Methods* 4 84–99.

[B48] Mehrdad A. Farhad S. Maryam B. (2011). The effects of problem solving skills training on test anxiety among college students. *Dev. Psychol. J. Iran. Psychol.* 8 67–74.

[B49] Mendonca M. (2001). Preparing for ethical leadership in organizations. *Can. J. Administr. Sci.* 18 266–276. 10.1111/j.1936-4490.2001.tb00262.x

[B50] Milfont T. L. Fischer R. (2010). Testing measurement invariance across groups: applications in crosscultural research. *Int. J. Psychol. Res.* 3 111–121.

[B51] Mumford M. D. Zaccaro S. J. Harding F. D. Jacobs T. O. Fleishman E. A. (2000). Leadership skills for a changing world: solving complex social problems. *Leadersh. Q.* 11 11–35.

[B52] Murphy S. E. (1992). *The Contribution of Leadership Experience and Self-Efficacy to Group Performance Under Evaluation Apprehension.* Washington: University of Washington.

[B53] Muthén B. Kaplan D. (1985). A comparison of some methodologies for the factor analysis of non-normal likert variables. *Br. J. Math. Statist. Psychol.* 38 171–189. 10.1111/j.2044-8317.1985.tb00832.x

[B54] Muthén L. K. Muthén B. O. (1998–2010). *Mplus Use”s Guide*, 6th Edn. Los Angeles, CA: Muthén & Muthén 10.1111/j.2044-8317.1985.tb00832.x

[B55] Oltedal S. Garratt A. Bjertns O. Bjornsdottir M. Freil M. Sachs M. (2007). The NORPEQ patient experiences questionnaire: data quality, internal consistency and validity following a Norwegian inpatient survey. *Scand. J. Public Health* 35 540–547. 10.1080/14034940701291724 17852989

[B56] Orpinas P. (2010). “Social competence,” in *The Corsini Encyclopedia of Psychology*, 4th Edn Vol. 4 eds Weiner I. B. Craighead W. E. (Hoboken, NJ: Wiley), 1–2.

[B57] Page D. Wong P. T. P. (2000). “A conceptual framework for measuring Servant Leadership,” in *The Human Factor in Shaping the Course of History and Development*, ed. Adjibolooso S. B. S. K. (Lanham, MD: University Press of America), 69–109.

[B58] Park G.-P. (2014). Factor analysis of the foreign language classroom anxiety scale in korean learners of english as a foreign language. *Psychol. Rep.* 115 261–275. 10.2466/28.11.PR0.115c10z2 25153961

[B59] Patel B. (2012). *The Importance of Resilience in Leadership*. London: Clore Social Leadership.

[B60] Rhemtulla M. Brosseau-Liard P. É and Savalei V. (2012). When can categorical variables be treated as continuous? A comparison of robust continuous and categorical SEM estimation methods under suboptimal conditions. *Psychol. Methods* 17 354–373. 10.1037/a0029315 22799625

[B61] Rolstad S. Adler J. Rydén A. (2011). Response burden and questionnaire length: Is shorter better? A review and meta-analysis. *Value Health* 14 1101–1108. 10.1016/j.jval.2011.06.003 22152180

[B62] Russell R. F. Stone A. G. (2002). A review of servant leadership attributes: Developing a practical model. *Leadersh. Organ. Dev. J.* 23 145–157. 10.1108/01437730210424

[B63] Schmitt N. Kuljanin G. (2008). Measurement invariance: review of practice and implications. *Hum. Res. Manag. Rev.* 18 210–222. 10.1016/j.hrmr.2008.03.003 17892098

[B64] Schneider B. Ehrhart M. G. Mayer D. M. Saltz J. L. Niles-Jolly K. (2005). Understanding organization-customer links in service settings. *Acad. Manag. J.* 48 1017–1032. 10.5465/amj.2005.19573107

[B65] Schneider B. White S. S. Paul M. C. (1998). Linking service climate and customer perceptions of service quality: test of a causal model. *J. Appl. Psychol.* 83 150–163. 10.1037/0021-9010.83.2.150 9577232

[B66] Sendjaya S. Sarros J. C. (2002). Servant leadership: Its origin, development, and application in organizations. *J. Leadersh. Organ. Stud.* 9 57–64. 10.1177/107179190200900205

[B67] Shek D. T. L. Chai W. Y. (2019). Psychometric properties of the service leadership attitude scale in hong kong. *Front. Psychol*. 10:1070. 10.3389/fpsyg.2019.01070 31133950 PMC6524405

[B68] Shek D. T. L. Chung P. P. Y.(eds) (2015). *Promoting Service Leadership Qualities in University Students* (1st Edn.). Singapore: Springer.

[B69] Shek D. T. L. Chung P. P. Y. Leung H. (2015a). How unique is the service leadership model? A comparison with contemporary leadership approaches. *Int. Disabil. Hum. Dev*. 14 217–231.

[B70] Shek D. T. L. Chung P. P. Y. Leung H. (2015b). Manufacturing economy vs. service economy: implications for service leadership. *Int. J. Disabil. Hum. Dev.* 14 205–215.

[B71] Shek D. T. L. Chung P. P. Y. Lin L. Leung H. Ng E. C. W. (2018a). “Service Leadership under the Service Economy,” in *Global and Culturally Diverse Leaders and Leadership*, 1st Edn, eds Chin J. L. Trimble J. E. Garcia J. E. (Bingley: Emerald Publishing), 143–161.

[B72] Shek D. T. L. Ma L. K. Lin L. Leung H. (2018b). Psychometric properties of the service leadership behavior scale: preliminary findings. *Int. J. Child Adolesc. Health* 11 427–443.

[B73] Shek D. T. L. Ma L. K. Ma M. S. C. Hoshmand R. A. (2018c). Convergent and factorial validation of the service leadership behavior scale [Special issue]. *Int. J. Child Adolesc. Health* 11 479–492.

[B74] Shek D. T. L. Ma L. K. Yu L. Leung L. M. (2018d). Validation of the service leadership knowledge scale: factorial and convergent validity [Special issue]. *Int. J. Child Adolesc. Health* 11 455–466.

[B75] Shek D. T. L. Zhu X. Chan K.-M. (2018e). Development of service leadership behavior scale: background and conceptual model [Special issue]. *Int. J. Child Adolesc. Health* 11 415–424.

[B76] Shek D. T. L. Zhu Y. F. A. Ma K. L. Lin L. (2018f). Validation of the service leadership attitude scale in hong kong [Special issue]. *Int. J. Child Adolesc. Health* 11 467–477. 10.3389/fpsyg.2019.01070 31133950 PMC6524405

[B77] Shek D. T. L. Chung P. P. Y. Lin L. Merrick J. (2017). *Service Leadership Education for University Students*, eds Edn. New York, NY: Nova Science.

[B78] Shek D. T. L. Chung P. P. Y. Yu L. Merrick J. (2015c). Service leadership curriculum and higher education reform in hong kong [Special issue]. *Int. J. Disabil. Hum. Dev.* 14 297–306.

[B79] Shek D. T. L. Leung H. (2015). “Service Leadership qualities in university students through the lens of student well-being,” in *Promoting Service Leadership Qualities in University Students* (1st Edn, eds Shek D. T. L. Chung P. P. Y. (Singapore: Springer), 1–16. 10.1007/978-981-287-515-0_1

[B80] Shek D. T. L. Li X. (2015). The role of a caring disposition in Service Leadership. *Int. J. Disabil. Hum. Dev.* 14 319–332.

[B81] Shek D. T. L. Lin L. (2015a). Core beliefs in the service leadership model proposed by the hong kong institute of service leadership and management. *Int. J. Disabil. Hum. Dev.* 14 233–242.

[B82] Shek D. T. L. Lin L. (2015b). “Evaluating Service Leadership programs with multiple strategies,” in *Promoting Service Leadership Qualities in University Students* (1st Edn, eds Shek D. T. L. Chung P. P. Y. (Singapore: Springer), 197–211. 10.1007/978-981-287-515-0_13

[B83] Shek D. T. L. Lin L. (2015c). Intrapersonal competencies and service leadership. *Int. J. Disabil. Hum. Dev.* 14 255–263.

[B84] Shek D. T. L. Lin L. (2015d). Leadership and mentorship: service leaders as mentors of the followers. *Int. J. Disabil. Hum. Dev.* 14 351–359.

[B85] Shek D. T. L. Lin L. (2017). “Validation of the Service Leadership Knowledge Scale: Criterion-related validity,” in *Service Leadership Education for University Students*, Eds. Edn, eds Shek D. T. L. Chung P. P. Y. Lin L. Merrick J. (New York NY: Nova Science), 189–204.

[B86] Shek D. T. L. Ma C. M. S. (2010). Dimensionality of the chinese positive youth development scale: confirmatory factor analyses. *Soc. Indic. Res.* 98 41–59. 10.1007/s11205-009-9515-9

[B87] Shek D. T. L. Ma C. M. S. (2014). Validation of a subjective outcome evaluation tool for participants in a positive youth development program in hong kong. *J. Pediatr. Adolesc. Gynecol.* 27(Suppl.), S43–S49. 24792762 10.1016/j.jpag.2014.02.011

[B88] Shek D. T. L. Yu L. (2014). Factorial validity of a subjective outcome evaluation tool for implementers of a positive youth development program. *J. Pediatr. Adolesc. Gynecol.* 27 S32–S42. 10.1016/j.jpag.2014.02.010 24792761

[B89] Snell R. S. Chan M. Y. L. Zou T. X. P. (2017). “Key practices of leadership for service in Hong Kong,” in *Service Leadership Education for University Students*, eds Shek D. T. L. Chung P. P. Y. Lin L. Merrick J. (New York, NY: Nova Science), 127–138.

[B90] Swami V. Barron D. Weis L. Voracek M. Stieger S. Furnham A. (2017). An examination of the factorial and convergent validity of four measures of conspiracist ideation, with recommendations for researchers. *PLoS One* 12:e0172617. 10.1371/journal.pone.0172617 28231266 PMC5322923

[B91] Towers Watson (2012). *The Next High-Stakes Quest: Balancing Employer and Employee Priorities— 2012-2013 Global Talent Management and Rewards Study*. Available at: https://www.towerswatson.com/en-HK/Insights/IC-Types/Survey-Research/Results/2012/09/2012-Global-Talent-Management-and-Rewards-Study (accessed April 21, 2018).

[B92] University Grants Committee [UGC]. (2017). *Student Enrolment of UGC-funded Programmes by University, Level of Study, Mode of Study and Sex, 2010/11 to 2016/17*. Hong Kong: UGC.

[B93] van de Schoot R. Lugtig P. Hox J. (2012). A checklist for testing measurement invariance. *Eur. J. Dev. Psychol.* 9 486–492. 10.1080/17405629.2012.686740

[B94] Wielkiewicz R. M. (2000). The leadership attitudes and beliefs scale: an instrument for evaluating college students’ thinking about leadership and organizations. *J. Coll. Stud. Dev.* 41 335–347.

[B95] Wong A. Liu Y. Tjosvold D. (2015). Service leadership for adaptive selling and effective customer service teams. *Ind. Mark. Manag.* 46 122–131. 10.1016/j.indmarman.2015.01.012

[B96] Wong P. T. P. Page D. (2003). *Servant Leadership: An Opponent-Process Model and the Revised Servant Leadership Profile*. Available at: https://www.regent.edu/acad/global/publications/sl_proceedings/2003/wong_servant_leadership.pdf (accessed July 31, 2018).

[B97] Wu H.-C. Mohi Z. (2015). Assessment of service quality in the fast-food restaurant. *J. Foodserv. Bus. Res.* 18 358–388. 10.1080/15378020.2015.1068673

[B98] Young J. K. Beaujean A. A. (2011). Measuring personality in wave I of the national longitudinal study of adolescent health. *Front. Psychol.* 2:158. 10.3389/fpsyg.2011.00158 21808628 PMC3139206

[B99] Yukl G. Seifert C. F. Chavez C. (2008). Validation of the extended influence behavior questionnaire. *Leadersh. Q.* 19 609–621. 10.1016/j.leaqua.2008.07.006

